# Preoptimisation in patients with acute obstructive colon cancer (PREOCC) – a prospective registration study protocol

**DOI:** 10.1186/s12876-023-02799-z

**Published:** 2023-05-25

**Authors:** Jeske R. E. Boeding, Paul D. Gobardhan, Arjen M. Rijken, Tom C. J. Seerden, Cornelis Verhoef, Jennifer M. J. Schreinemakers

**Affiliations:** 1 Department of Surgery, Amphia Hospital, Breda, the Netherlands; 2 Department of Surgical Oncology, Erasmus MC Cancer Institute, Rotterdam, The Netherlands; 3 Department of Gastroenterology, Amphia Hospital, Breda, the Netherlands

**Keywords:** Obstruction, Colon cancer, Optimisation, Mortality, Morbidity, Oncological outcome

## Abstract

**Background:**

Postoperative mortality and morbidity rates are high in patients with obstructing colon cancer (OCC). Different treatment options have been evaluated over the years, mainly for left sided OCC. Optimising the preoperative health condition in elective colorectal cancer (CRC) treatment shows promising results. The aim of this study is to determine whether preoptimisation is feasible in patients with OCC, with a special interest/focus on right-sided OCC, and if, ultimately, optimisation reduces mortality and morbidity (stoma rates, major and minor complications) rates in OCC.

**Methods:**

This is a prospective registration study including all patients presenting with OCC in our hospital. Patients with OCC, treated with curative intent, will be screened for eligibility to receive preoptimisation before surgery. The preoptimisation protocol includes; decompression of the small bowel with a NG-tube for right sided obstruction and SEMS or decompressing ileostomy or colostomy, proximal to the site of obstruction, for left sided colonic obstructions. For the additional work-up, additional nutrition by means of parenteral feeding (for patients who are dependent on a NG tube) or oral/enteral nutrition (in case the obstruction is relieved) is provided. Physiotherapy with attention to both cardio and muscle training prior surgical resection is provided. The primary endpoint is complication-free survival (CFS) at the 90 day period after hospitalisation. Secondary outcomes include pre- and postoperative complications, patient- and tumour characteristics, surgical procedures, total in hospital stay, creation of decompressing and/or permanent ileo- or colostomy and long-term (oncological) outcomes.

**Discussion:**

Preoptimisation is expected to improve the preoperative health condition of patients and thereby reduce postoperative complications.

**Trial registration:**

Trial Registry: NL8266 date of registration: 06-jan-2020.

**Study status:**

Open for inclusion.

**Supplementary Information:**

The online version contains supplementary material available at 10.1186/s12876-023-02799-z.

## Background

Mortality and morbidity after surgery for obstructing colon cancer (OCC) occurs more frequently compared to patients without obstruction. [1–3] OCC is often treated with emergency resection, which is associated with high mortality and morbidity rates. [3–7] To avoid an emergency resection, multiple treatment options have been evaluated over the years. [8–17].

Focussing on resolving the obstruction in the short term and postponing the resection itself, could create an important time frame, providing a chance to optimise the medical condition of the patient, allowing for a complete preoperative screening of the patient’s health status and examine possible concomitant illnesses in patients with OCC. This window of opportunity allows for optimisation of the nutritional status and functional capacity in patients with OCC before surgical resection. Both malnutrition and the patient’s functional capacity seem to be influential factors in postoperative mortality and morbidity. [18–21] Encouraging results have been described for postoperative recovery after implementing prehabilitation in elective abdominal and colorectal operations. [22–28] Although large trials have not yet been conducted to this day, the first data of preoptimisation in right-sided OCC show promising results. In addition, prehabilitation is becoming an increasingly popular method.

To further analyse the influence of pre-operative optimisation in patients with OCC, a prospective registration study was initiated.

### Study objectives

The aim of this study is to further analyse preoptimisation in patients with OCC, with a special interest/focus on right-sided OCC. Furthermore, this study investigates whether preoptimisation before resection improves postoperative mortality and morbidity in OCC. The preoptimisation protocol includes supplementary nutrition (total parenteral nutrition (TPN) or tube feeding), physiotherapy before surgery, and, if needed, bowel decompression. At the start of this prospective registration study, no comparable studies or centres performed preoptimisation in patients diagnosed with OCC.

## Methods

### Study design

This is a single centre prospective registration study, which administers optimisation before resection to all patients diagnosed with OCC in our hospital treated in curative intent for OCC. The presentation of OCC patients in this study is that of imminent or complete obstruction caused by OCC. The purpose is to improve the clinical condition in patients with small bowel ileus caused by the OCC. The goal is improving the clinical condition through decompression of the small bowel, restoring fluid- and electrolyte balance, improving nutritional status through parenteral feeding and physical therapy during the clinical period which is focused on reduction of muscle wasting and if possible muscle gaining. The preoptimisation protocol focuses on right-sided OCC, but also includes left-sided OCC with a slight deviation in terms of bowel decompression technique. A written informed consent is obtained from all patients.

### Study population and eligibility

Participants will be recruited from the Amphia Hospital, Breda the Netherlands. All patients (≥ 18 years of age) with a high suspicion of, or histologically proven, colon cancer that are diagnosed with imminent or complete obstruction will be included. To be eligible to participate in this study, patients need to meet the following inclusion criteria: 1) obstruction is caused by colon cancer (whether of high suspicion radiologically or histologically proven) and 2) symptoms of obstruction (abdominal pain, nausea, vomiting, and diarrhoea) confirmed by the presence of a dilated colon or ileum on computed tomography (CT) scan. Excluded from the study are all patients with 1) obstruction of the colon pathologically, caused by benign disease; 2) obstruction caused by an extra colonic malignancy; 3) emergency complications (sepsis, peritonitis, perforation by tumour or blow out) diagnosed at presentation; or 4) rectal cancer.

A specialised gastrointestinal surgeon/oncological surgeon determines if preoptimisation is safe and whether or not the patient is eligible. Patients that are diagnosed with signs of sepsis, haemodynamic instability, perforation of the bowel or suspicion of bowel ischaemia or other reasons for which immediate surgery is required, are excluded for optimisation. In case of high suspicion of OCC radiologically, patients may receive a colonoscopy to confirm cancer preoperatively (if eligible). A colonoscopy is not routinely performed in right sided obstruction because of the imminent / complete obstruction and because the use of SEMS has not been well studied in these patients we will rely on the NG to decompress the more proximal colon. Final histology of the specimen will be used to confirm the diagnosis after resection. In case of left sided OCC a colonoscopy is often routinely performed either to achieve decompression by stent placement or after decompression by an ileostomy of colostomy and is beneficiary for the diagnosis of the actual malignancy by visualisation of the tumour and biopsy.

If the patient is eligible, optimisation before surgery is started according to the preoptimisation protocol. It is of the utmost importance to ensure decompression of the small bowel and if present the colon. Special care should be given to adequate decompression in case a competent ileocecal valve hampers decompression by the nasogastric tube. For instance in a hepatic flexure tumour, decompression of the ascending colon is also desired to avoid complications like a perforation and/or rupture. Therefore, next to placement of the NG tube, clinical evaluation by a physician and monitoring of the NG tube output is mandatory. All patients admitted to the surgical ward, starting with the optimisation protocol, are revaluated by the responsible physician within several hours after admission. If there is any doubt of adequate decompression additional imaging (X-ray or CT-scan) will be performed. In case of clinical deterioration, discomfort and/or increase of leukocyte count and C-reactive protein is early surgery, or alternative decompression or emergency resection is considered (with or without additional imaging preoperatively). No specific considerations are made for the determination whether or not the ileocecal valve is competent. The management is the same regardless of this finding, namely daily mandatory clinical examination, laboratory tests and the preoptimisation nutrition and physical therapy.

All patients are discussed in a multidisciplinary meeting in order to provide an accurate treatment approach towards surgery, neo-adjuvant and/or adjuvant chemotherapy as well as postoperative treatment options. After preoptimisation, definite resection is performed at least 7 days after initial decompression and no later than 4 weeks after initial presentation, unless gastrointestinal surgeons/oncological surgeons have reasons to perform an acute resection. To provide a good overview of all treatment options used in patients with OCC, we will prospectively report all patients treated for OCC. Meaning that all patients, even if not eligible for preoptimisation, will be asked to participate for prospective data registration.

### Study procedure

**Fig. 1:**
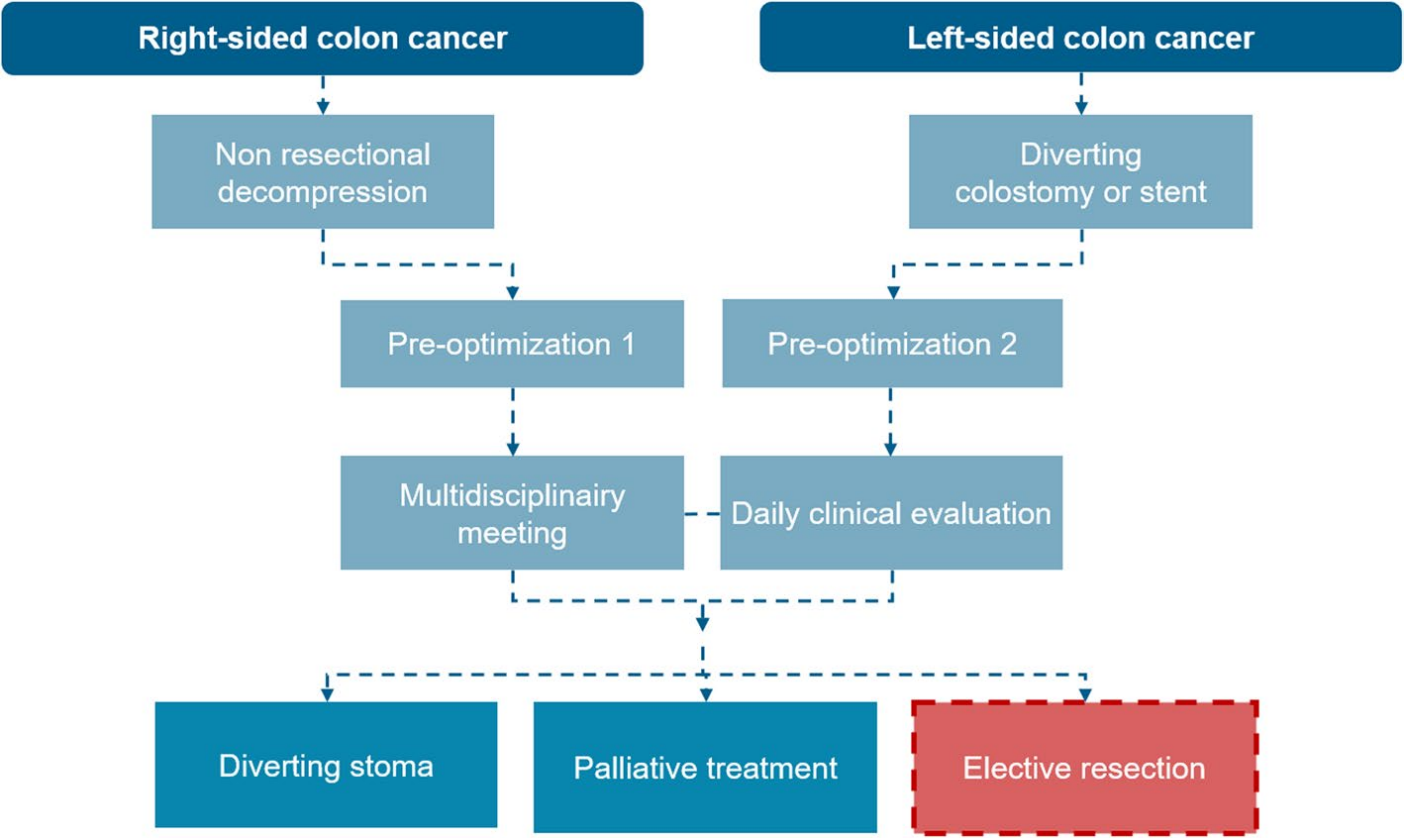
Flow chart of treatement strategies obstructing colon cancer

**Fig. 2:**
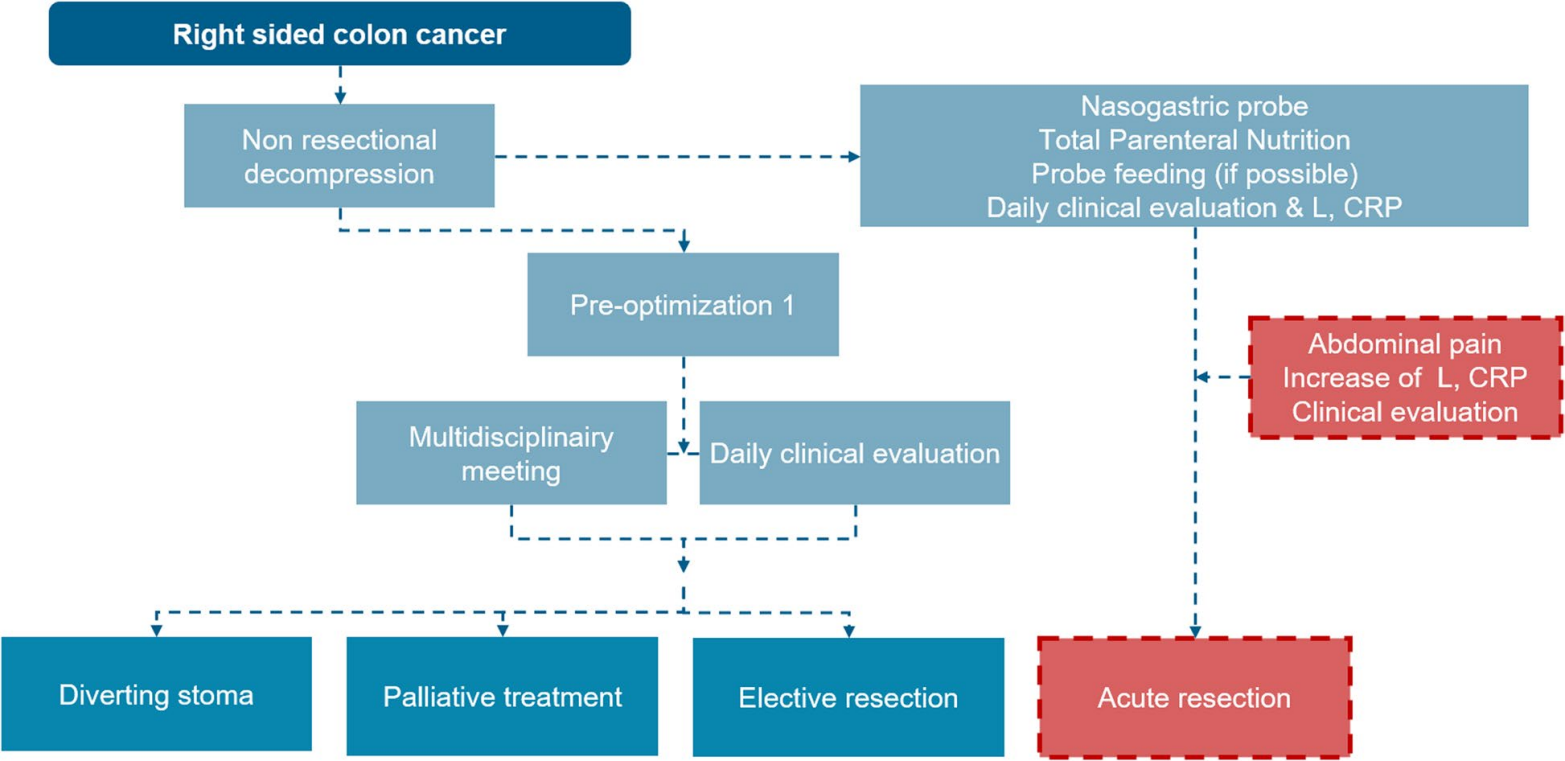
Flow chart right-sided colon cancer and treatment strategies during optimisation

Patients eligible for treatment, will receive preoptimisation and decompression, followed by scheduled surgical resection. Preoptimisation of patients will be divided into preoptimisation 1 (right-sided OCC) and preoptimisation 2 (left-sided OCC). (Figs. 1 and 2).

#### Preoptimisation 1: RIGHT-sided OCC

**Fig. 3:**
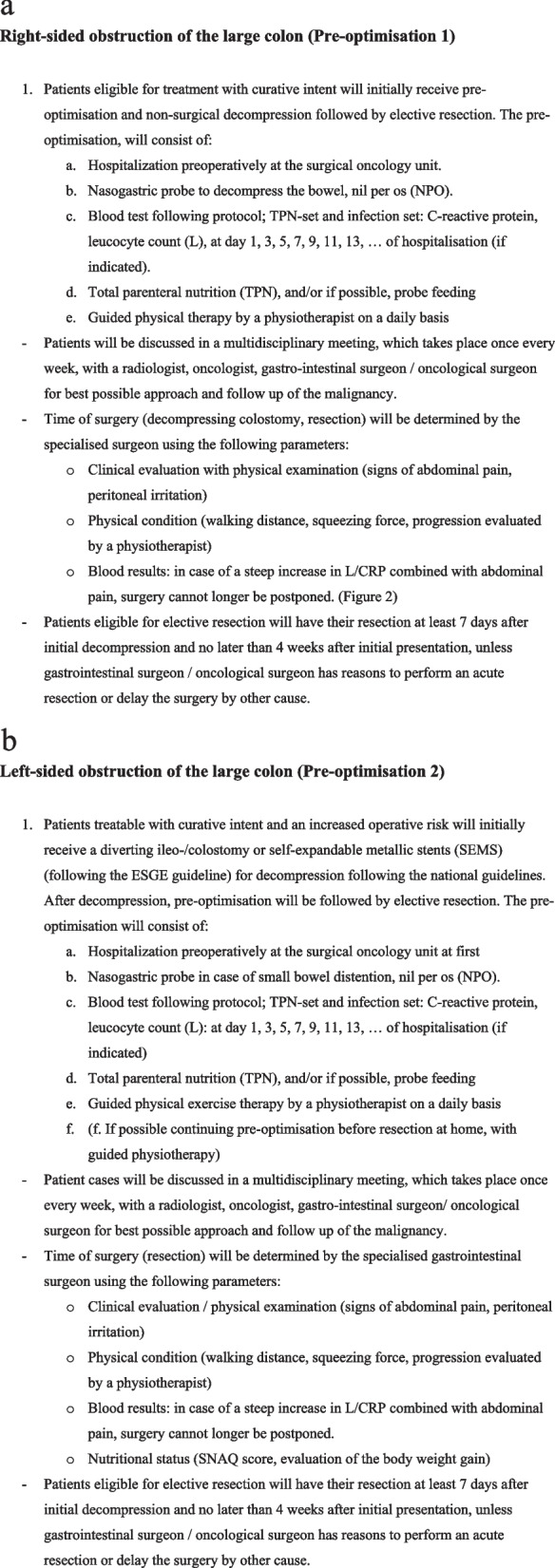
**a** Optimisation protocol for right-sided colon cancer. **b** Optimisation protocol for left-sided colon cancer

Patients with right-sided OCC will receive preoptimisation according to the protocol shown in Fig. 3a. This protocol consists of hospitalisation, nonsurgical decompression of the bowel by a nasogastric tube, supplementary nutrition by total parenteral nutrition (TPN) or enteral feeding by nasogastric tube. Nutritional status is evaluated at the surgical department with the use of Short Nutritional Assessment Questionnaire (SNAQ) score. [29] In case of malnutrition, a dietician is routinely consulted to provide an additional nutritional plan to optimise the nutritional status. A substantial calibre of nasogastric tube (≥ 16 Fr) is placed, in order to attain sufficient decompression of the bowel. During the preoperative period, the patient is monitored daily for clinical deterioration. Specific attention is given to the distention of the abdomen and comfort. In case of progression of discomfort, first the nasogastric tube is evaluated for its function. In case of clinical deterioration the inflammatory parameters are measured, leucocyte count and C- reactive protein. Patients are evaluated daily, which includes physical examinations, physical conditions, blood results (every other day) and nutritional condition. In addition vital parameters of the patients will be assessed several times a day. Routine clinical evaluation and alleviation of the pain experienced by the patient after decompression, indicates adequate decompression. However, in case of a steep increase in the leukocyte count (L) and/or C-reactive protein (CRP) level, combined with abdominal pain and/or clinical deterioration, early surgery will considered. During hospitalisation, patients will receive daily guided physiotherapy and exercises to improve their physical condition. Elective resection will be performed at least seven days after initial decompression, and no later than four weeks after initial presentation, unless the gastrointestinal surgeon/oncological surgeon has reasons to perform an acute intervention/resection (as described above).

#### Preoptimisation 2: LEFT-sided OCC

Patients with left-sided OCC will receive optimisation according to the protocol shown in Fig. 3b, consisting of nonsurgical decompression of the bowel by nasogastric tube (if necessary), diverting ileostomy or colostomy proximal to the site of obstruction, or self-expandable metallic stents (SEMS) placement as a bridge to surgery. The European Society of Gastrointestinal Endoscopy (ESGE) recommends stenting as a bridge to surgery to be discussed, within a shared decision-making process, as a treatment option in patients with potentially curable left-sided obstructing colon cancer as an alternative to emergency resection. [30] Nutritional condition is reported at the surgical department with the use of Short Nutritional Assessment Questionnaire (SNAQ) score. [29] In case of malnutrition, a dietician is consulted to provide an additional nutritional plan. Supplementary nutrition includes feeding by enteral feeding by nasogastric tube/total parenteral nutrition. Patients are evaluated daily, inclusive of physical examinations, physical conditions, blood results (every other day) and nutritional condition. In the case of a steep increase in the leukocyte count (L) and/or C-reactive protein (CRP) level, combined with abdominal pain and/or clinical deterioration, surgery can no longer be postponed. During hospitalisation, patients will receive guided physiotherapy and exercises to improve their physical treatment, which would ultimately be evaluated and registered by the physiotherapist. If bowel continuation is restored after colostomy/or stenting, oral and/or enteral feeding will be resumed. Optimisation will be continued from home (if feasible) until scheduled resection is performed. Elective resection will be performed at least 7 days after initial decompression, and no later than four weeks after initial presentation, unless the gastrointestinal surgeon/oncological surgeon has reasons to perform an acute intervention/resection (as described above).

#### Nutrition

Patients diagnosed with OCC often have a poor preoperative health condition and/or electrolyte deficits. The nutritional status differs for each individual patient depending on the length of symptoms of obstruction, presentation, and other patient characteristics. The SNAQ score is evaluated at the surgical department and a dietician is consulted. The complete nutritional assessment will be performed by a dietician on the day of diagnosis to specify the patient’s nutritional condition. Additional nutrition is supplemented prior to surgery according to the standard hospital protocols. Any patient with negligible food intake for more than five days is at risk of developing refeeding problems. This means that in patients with high risk of refeeding syndrome, an adapted refeeding schedule needs to be utilised and that the maximal energy requirements are only met at day 3–4 after starting parenteral nutrition. In this case, an additional 6–7 days of preoptimisation needs to be completed. Postoperatively, patients will receive additional nutrition in case this is deemed necessary. Enteral feeding is considered in patients diagnosed with imminent obstruction, for example with low residual enteral feeding by nasogastric tube if possible. Patients body weight is monitored from the start of the study following a 90-day period.

During hospital admission, current medication will be switched from oral to intravenous administration. When this is not possible, an alternative medication will be subscribed, or the medication will be stopped until further notice when deemed safe.

#### Physical therapy

In patients diagnosed with OCC, clinical deterioration often prevents them from physical (strenuous) sports activity. Following the preoptimisation protocol, patients are encouraged to improve their physical condition, in order to prevent wastage of muscle. All patients that are admitted to the hospital will be evaluated by a physiotherapist. Patients undergo custom-made supervised in-hospital training sessions once per day during their hospital stay. Additionally, patients are encouraged to do exercises themselves during their hospital stay. The nursing staff has been instructed and educated to encourage patients and family members to train and exercise if possible in case repeated regular supervision is not possible. If a patient is unable to complete the exercise schedule, a physiotherapist evaluates the possibilities and the intensity is reduced by 10%. The intensity is reduced further – in steps of 10%—until patients can complete the programme. During training sessions, strength training is conducted every other day.

Patients will also be given instructions about how to conduct aerobic exercises during their hospital stay unsupervised. Patients are instructed to aim for 45 min walking and/or cycling a day, with a minimum of 30 min a day. This can be less when patients have low capacity due to their deteriorated physical and nutritional condition. If possible, patients can do more than 45 min of walking or cycling every day. In case of a low exercise capacity, it is advised to walk/cycle 2–3 times a day for periods of 10–20 min. Also a stationary bicycle and/or a walking aid (walker) is allowed and even encouraged. Breathing techniques, with an incentive spirometer, are also implemented to prevent pneumonia, in the goal to hopefully optimise patient health condition prior to surgical resection.

If optimisation can be performed outside of the hospital, for instance after SEMS placement or decompressing –ostomy, patients will receive a varied training program which will be supervised by physiotherapist in the hospital or with a physiotherapist by choice.

#### Palliative treatment

Patients diagnosed with OCC with no curable options, including (extensive) metastatic disease, will be treated in palliative care. Bowel decompression, if needed, will be performed with SEMS placement, or diverting ileo-/colostomy. The decision to do palliative treatment is confirmed in the weekly multidisciplinary meeting, which includes all treating physicians.

### Data management

#### Data extraction

Baseline characteristics that will be collected at presentation, are shown in Table 1. These characteristics are common care for patients presenting with abdominal complaints in our emergency room. The physician in care is responsible for medical history and physical examination. Laboratory results are evaluated by the physician in care. Before addition to the surgical department, the pharmacist’s assistant will check the patient’s medications. During admission, tests will be repeated if indicated following the protocol. All measurements (e.g. laboratory results, total days of TPN, nasogastric tube, time until first bowel movement, total stay in hospital (days) and complications) are retracted from the medical file of the patient by the project leader/ research assistant. (Table 1) Tumour characteristics, adjuvant treatment, definite stoma rates and outcome measurements, will be assembled by the research assistant/project leader in the hospital. The follow up period of the patients will not be altered, and normal follow-up is continued for CRC postoperatively (3/6/9/12 months).

**Table 1:** Study parameters and baseline characteristics that are collected in this study

Patients characteristics	gender, age at surgery—American Society of Anaesthesiologists (ASA) score—all co-morbidities—patient history (previous surgery, smoking status, allergies and current medications)—preoperative weight loss at 3 and 6 months before presentation (SNAQ score)—bodyweight at hospital presentation – height (centimetres)—bodyweight before surgery—blood pressure—nutritional risk (NRS)—concomitant and previous therapy (chemotherapy/radiation)—early warning score (EWS)
Laboratory values	sedimentation—C-reactive protein (CRP)—haemoglobin (Hb)—haematocrit (Ht) – leukocytes—prothrombin time (PTT)—renal function (sodium, potassium, glomerular filtration rate)—liver function (albumin, bilirubin, Alanine-Amino-Transferase (ASAT), Aspartate-Amino -transferase (ALAT), lactic acid dehydrogenase (LDH), alkaline phosphatase and gamma-GT) – creatinekinase -phosphate – lactate – vitamin D
Tumour characteristics	cancer stage (clinical and pathological) according to the tumour node metastasis (TNM) classification of the American Joint Committee – localization – metastases—endoscopic surveillance—tumour type – obstruction – pre-operative diagnostics (endoscopy, CT-scan, MRI, X-ray)
Surgical characteristics	surgical intervention—time till surgery (days)—pre-operative antibiotics—type of resection—laparoscopic/open procedure—diverting- or end colostomy—blood loss during surgery—operating time –oncological/gastrointestinal surgeon – time of surgery (day/night) – conversion – bowel distention operatively – serosa tears
During hospitalization	Nutrition (TPN/extra nutrition) – consultation of other specialist – pre-operative interventions (ultrasound, radiological interventions, medication needed) – weight gain – laboratory values – substantiation and decision for operation date
Post-operative	Intensive care surveillance – time nasogastric tube – time till bowel passage – complications (following Clavien-Dindo classification) – adjuvant treatment – hospitalisation days – interventions – radiology images
Follow up (3/6/912 months)	X-thorax – CEA – ultrasound of the liver – additional analysis if needed – weight loss/gain – metastasis – recurrence – endoscopy at 1 year FU

#### Outcome parameters

All data is recorded in Castor EDC prospectively which is only accessible by the investigators. The primary endpoint in this study is complication-free survival (CFS) at 90 days after hospitalisation. Complication is defined as mortality and/or development of a major complication (Clavien-Dindo classification > 2). With a total follow up period of one year. Secondly, all other complications, patient and tumour characteristics, surgical procedures/hospitalisation, permanent ileostomy or colostomy proximal to the site of obstructionas well as long-term (oncological) outcomes will be assessed.

Complications are scored by the Clavien-Dindo classification. [31] This classification can be used to correct a specific complication or to rank a complication in an objective and reproducible manner. Complications with a Clavien-Dindo score > 2 will be defined as a major complication. A standardised format for scoring complications will allow researchers to define the complications and treatment (Additional file 1).

#### Sample size calculation

We retrospectively analysed all patients that presented with acute obstructing CRC, between February 2011 and November 2015, in our hospital. A total of 114 patients presented with acute obstructing CRC, of which fourteen patients were diagnosed with rectal carcinoma. Eleven patients were treated with palliative surgery, and in eight patients, a blowout was reported. After exclusion of the above, a total of 80 patients were treated with curative intent. Acute resection was performed in 70 of the patients. In twenty-two patients (31%), our primary endpoint was not observed.

The primary outcome is a complication-free survival (CFS) at 90 days after hospitalisation. Sample size calculation is based on a one-sample binomial test of CFS, considering that the CFS probability is unacceptable below 70%, and is clinically meaningful above 85%. The 70% boundary is our conjecture of the 90-day CFS probability in patients treated according to common practice in our hospital. After applying our preoptimisation protocol, we expect this probability to increase towards 85%. Using a test size α of 0.05 (2-sided), this increase, from 70 to 85%, was detectable with 90% power in 90 patients. To account for the decrease in the number of patients at risk during follow-up, we will increase the sample size by approximately 20%, therefore to 110 patients.

### Statistical analysis

#### Primary study parameters

Statistical analysis will be performed using IBM SPSS Statistics Program version 25 and R-studio version 1.4.1717. The primary endpoint is complication-free survival (CFS) at 90 days after hospitalisation. Complication is defined here as a major complication (Clavien-Dindo classification > 2). So the combined endpoint considered here is either the first incidence of a major complication (that may include death) or death form possibly other causes while still being complication free.

#### Secondary study parameters

Cox’s Proportional Hazards regression analysis will be used to examine the effect of explanatory variables on CFS at 90 days and 1 year, and the possible modification of this effect after 90 days of follow up. The following candidate explanatory variables will be considered: creation of primary anastomosis, stoma creation, radical tumour resection, total in hospital stay and oncological outcome.

#### Other study parameters

Descriptive variables will be retrieved from the medical charts with a total follow up period of 1 year. (Table 1) Cox’s Proportional Hazards regression analysis will be used to examine the effect of explanatory variables on CFS at 90 days and 1 year, and the possible modification of this effect after 90 days of follow up.

#### Protocol and registration

This prospective registration study, analysing the optimisation protocol, did not require a DSMB committee while the optimisation protocol is already standard practice in this hospital. The study protocol is registered on Trial Registry: NL8266, date of registration: 06-jan-2020.

## Discussion

Management of patients with OCC is a challenge. Different treatment options, risk factors and improvements in postoperative care for OCC have been evaluated over the years. [4, 32–36] Treatment for OCC can be divided roughly into two main options: 1) emergency resection or 2) staged treatment (alleviation of the obstruction with secondary resection of the tumour). It has been shown that emergency resection has poor outcomes compared to elective colorectal surgery.

### Emergency resection & staged treatment

Emergency resection was the most commonly performed treatment for left-sided OCC. [6] However, in the Netherlands, there has been a paradigm shift away from emergency resection for left-sided OCC. This while emergency resection has been associated with an increased risk of postoperative mortality, morbidity and permanent stoma rates. Mortality rates after emergency surgery for OCC can increase up to 41% in elderly patients with multiple comorbidities. [2–4, 6, 16] In right sided OCC, the most commonly used treatment is still emergency resection, [37] despite the poor outcome on morbidity and mortality compared to patients treated electively. [4, 5, 32, 36, 38].

Different treatment strategies avoiding emergency resection in OCC (e.g. loop colostomy, stent placement and tube decompression) have been mainly analysed for left-sided OCC. [17, 37]. Over the years, positive results for left-sided OCC treated with decompressing ileostomy or self-expandable metallic stents (SEMS) as a bridge to surgery, such as improved short-term mortality and morbidity compared to emergency surgery have been reported. [16, 39, 40] For right-sided OCC the literature is far less extensive, compared to left-sided OCC. However the high postoperative mortality and morbidity rates after emergency surgery for right-sided OCC, compared to patients treated electively for CRC, indicate the need for alternative treatment options. Much room for improvement remains in patients with OCC.

### Optimisation

New insights show that the preoperative health status in elective CRC treatment is of importance to the postoperative outcome. [18–21] Promising data in postoperative recovery, after prehabilitation programs in elective abdominal and colorectal operations, have been released, as well as for other elective surgical procedures. [22–28] A recent Dutch trial by van Berkel et al. demonstrated the value of prehabilitation in elective colon resection while it reduces the risk of postoperative complications in high-risk patients. [41].

The worse clinical status in patients presenting with OCC is probably one of the main causes of the poor short-term compared to non-obstructing OCC. Obstruction of the colon is often accompanied by symptoms such as nausea, abdominal pain, and vomiting, leading to altered intake or no intake. This may lead to malnutrition and a poor physical health status at time of presentation. The preoperative timeframe, which is needed for preoptimisation, is often short or non-existent in patients with OCC. However, staged treatment to avert emergency surgery, creates a preoperative time frame providing a chance for optimisation of the patient’s medical condition before tumour resection and allowing to do a complete preoperative screening of the patient’s health status and examine possible concomitant illnesses. The influence of optimisation in OCC may be of great value in case of reducing postoperative complications, morbidity and mortality.

Refraining from emergency surgery in OCC can be a barrier for professionals because of concern for complications due to the distended (small) bowel and colon. Adequate management of the obstruction is crucial, while left untreated the obstruction could lead to bowel necrosis, perforation, and ultimately death. [42] However, the feasibility of different staged procedures has been demonstrated over the years, such as SEMS and decompressing ileo- or (transverse) colostomy for left-sided OCC. Non-surgical bowel decompression by nasogastric tube for (right-sided) OCC has not yet been proved in large studies. We realise that concerns may be raised over the strategy to decompress the small bowel and proximal colon in patients with an imminent or complete obstruction in OCC. Therefore, daily assessment of the patients with right-sided OCC in this study is crucial. In case a caecum dilation > 10 cm, decompression is immediately needed, while this extensive diameter is often accompanied by a competent ileocecal valve. Clinical and biochemical values need to be monitored carefully, and immediate interference is needed in case of abdominal pain, increase of leukocytes or C-reactive protein, to prevent bowel necrosis, perforation of a blowout. The feasibility of postponing surgery without decompressing stent or ileostomy as a bridge to surgery has recently be confirmed in a recent study of Fahim et al.. This study included all consecutive bowel obstruction patients treated with dietary adjustments, laxatives and prehabilitation before resection. In this study a total of 24 patients with bowel distention receiving TPN, reported emergency surgery in only 25% of the patients, while 75% of these patients were treated electively after 7–10 days of prehabilitation. [43] This study differs from this study protocol while they also included benign disease. However, the feasibility of postponing surgery without SEMS or decompressing ileostomy was showed. In addition to this study, a retrospective pilot study performed in the Amphia hospital confirmed the feasibility of postponing surgery as well. In a total of 16 patients presenting with right-sided OCC, bowel decompression using a nasogastric tube was performed and emergency interference was not needed.

Another barrier for postponing emergency resection in OCC may be the uncertainty concerning oncological outcome. This while postponing resection may lead to tumour treatment delay. However, recent studies in patients treated with SEMS as bridge to surgery in left-sided OCC showed that the oncological outcomes were comparable with those from emergency surgery. [44, 45] Even though the oncological outcome in optimisation of OCC patient is unknown, optimisation may influence postoperative outcome positively. While high rates of postoperative complications after emergency surgery may lead to prolonged hospital stay and time to recovery. [3, 7, 46] The increased recovery time may lead to delayed adjuvant chemotherapy treatment, or no adjuvant treatment at all which has been associated with significantly worse overall survival and a higher recurrence rate. [47].

The aim of this study is to determine whether optimisation in patients presenting with OCC is feasible, with special interest for right-sided OCC. We believe that this preoptimisation is beneficial for the majority of the patients. Next to a poor nutritional status and physical condition, it is often necessary to restore the fluid- and electrolyte balance. By adopting this strategy, there is time to improve these factors. Because of the acute setting, we do not think it is possible to define a homogeneous group that can be treated with preoptimisation or other valid treatment procedures (acute resection or diverting stoma). This is why randomisation, according to our opinion, is not a preferred option in our study.

## Supplementary Information

**Additional file 1. **

## Data Availability

Data sharing is not applicable to this article as no datasets were generated or analysed during the current study.

## Abbreviations

AL: Anastomotic leakage
BSE: Sedimentation rate erythrocytes
CI: Confidence interval
CCI: Comprehensive complication index
CRC: Colorectal cancer
CRP: C-reactive protein
CT: Computed tomography
DRCA: Dutch Surgical Colorectal Audit
EWS: Early warning score
HR: Hazard ratio
L: Leucocyte count
MEC-U: Medical Research Ethics Committees United
OCC: Obstructing colon cancer
SEMS: Self-expandable metal stent
TPN: Total parenteral nutrition
SNAQ: Short Nutritional Assessment Questionnaire
