# Supplementary material for: Preoptimisation in patients with acute obstructive colon cancer (PREOCC) – a prospective registration study protocol
Source: BMC Gastroenterol. 2023 May 25;23:186. doi: 10.1186/s12876-023-02799-z (PMC10214621; doi:10.1186/s12876-023-02799-z)
Supplement: Supplementary file 1 — Additional file 1. [file 12876_2023_2799_MOESM1_ESM.docx]

**Additional file 1**

**Clavien-Dindo Classification**

**Grade I:** Any deviation from the normal postoperative course without the need for pharmacological treatment or surgical, endoscopic and radiological interventions Allowed therapeutic regimens are: drugs as antiemetics, antipyretics, analgetics, diuretics and electrolytes and physiotherapy. This grade also includes wound infections opened at the bedside.

**Grade II:** Requiring pharmacological treatment with drugs other than such allowed for grade I complications. Blood transfusionsand total parenteral nutritionare also included.

**Grade III:** Requiring surgical, endoscopic or radiological intervention.

1. IIIa: Intervention not under general anesthesia
2. IIb: Intervention under general anesthesia

**Grade IV:** Life-threatening complication (including CNS complications)* requiring IC/ICU-management

1. IVa: Single organ dysfunction (including dialysis
2. IVb: Multiorgan dysfunction

**Grade V:** Death of a patient
